# Validity of the self-reported number of teeth in Chilean adults

**DOI:** 10.1186/s12903-019-0794-5

**Published:** 2019-06-04

**Authors:** Paula Margozzini, Rodrigo Berríos, Cynthia Cantarutti, Claudia Veliz, Duniel Ortuno

**Affiliations:** 1 Departamento de Salud Pública, Facultad de Medicina, Pontificia Universidad Católica de Chile, Santiago, Chile; 2 Escuela de Odontología, Facultad de Medicina, Pontificia Universidad Católica de Chile, Santiago, Chile

**Keywords:** Self-report, Oral examination, Number of teeth, Validity

## Abstract

**Background:**

Clinical dental evaluations are considered complex and costly measurements that epidemiological surveillance studies of multiple simultaneous chronic diseases currently require, for example National Health Surveys (ENS). Accordingly, simpler and more affordable methods need to be validated. The aim of this study was to assess the validity of the self-report on the total number of teeth in the general Chilean adult population.

**Methods:**

A substudy was conducted on ENS 2016–2017 participants. A stratified random sample of 101 of them was subjected to a telephone questionnaire. This information was then compared with the results obtained from the oral examination performed by a trained nurse during a home visit. Spearman correlations, intraclass correlation coefficients and the Bland-Altman method were used to analyse the data.

**Results:**

In men, the average number of teeth recorded during the oral examination coincided with the number of teeth in the self-report (22 teeth). In women, the total teeth average was 18 and 19 teeth according to the examination and self-report, respectively. For the total number of participants, a strong and significant Spearman correlation was obtained (ρ = 0.93); in men and women, the Spearman correlation observed was also strong and significant (ρ = 0.90 and ρ = 0.96 respectively). The value of the intraclass correlation coefficient indicated a significant concordance (CCI = 0.96) in both men and women (CCI = 0.93 and 0.98 respectively). A tendency to greater correlation was observed as the number of teeth decreased.

**Conclusions:**

The number of teeth self-reported by the subjects in this study correlated with the number of teeth recorded in the clinical examination. Self-report is a valid method to determine the number of teeth in national health surveys.

## Background

Oral diseases affect about half of the world’s population and their high impact is considered an important public health problem in terms of disease burden and treatment costs [1]. The main diseases that affect the oral cavity are caries and periodontal disease being both irreversible and cumulative conditions that may progress to tooth loss [2], which is the main cause of disease burden due to existing oral conditions in the world [1].

In addition to altering facial aesthetic parameters, absence of teeth or edentulism leads to masticatory function loss with negative repercussions on the nutritional status of the adult [3]. A lower number of teeth is considered a risk factor for systemic conditions such as coronary heart disease, peripheral vascular disease, heart failure and general mortality of cardiovascular origin [4]. Lastly, edentulism decreases the capacity for social interaction and the quality of life of individuals [5].

The number of remaining teeth determines the diagnosis of a functional dentition, defined as the presence of at least twenty permanent teeth in the mouth by the World Health Organization (WHO) [6]. However, oral functionality depends not only on the number of remaining teeth, but also on the masticatory efficiency and status of the soft and hard tissues in the mouth [7]. The literature indicates that shortened dental arches i.e. those that encompass the anterior and premolar teeth, meet the requirements of correct functionality [7]. The concepts of shortened dental arch and number of missing teeth are important variables for dental treatment decisions in partially edentulous patients [7]. Still the functional demands and the number of teeth required to meet such demands may vary between individuals [8].

In Chile, 19.4% of the estimated years lived with disability are caused by oral diseases [9]. The most important cause of tooth loss in the young population is caries whereas in the adult population it is chronic periodontal disease [10]. The absence of teeth increases with age; the population aged 35 to 44 years has 6.5 lost teeth on average while those aged 65 to 74 years average as many as 15.8 [9]. On the other hand, only 20% of adults aged 35 to 44 years retain their full dentition, while in individuals aged 65 to 74 years this prevalence decreases to 1% [11]. Additionally, in Chilean women aged 45 to 59 years, edentulism is the third specific cause of disease burden, being 2.8 times higher than in men [12]. A recent multinational study showed that regarding individuals older than 35 years, Chile had the lowest prevalence of edentulism, however, it had the highest adjusted proportion of individuals with less than 21 teeth [2]. The study concluded that edentulism has a high impact on the quality of life of Chileans [2].

When studying oral health status, one of the most commonly evaluated parameters is the number of teeth. The clinical dental examination is considered the *gold standard* method for this measurement [13] and the only source of valid information in the clinical study of oral diseases [14]; however, it has limitations associated with high costs in terms of personnel, time and resources necessary for its implementation in population studies [15]. Other disadvantages associated with the clinical examination include more time for execution, use of specialized materials, fatigue of examiners and increase in the likelihood of low response rates in studies [16]. In epidemiological surveillance, some efforts have been made to involve trained nurses in household studies for the general population, but this poses important additional efforts concerning standardization and training hours. Surveillance of non-communicable diseases in the general population requires simultaneous measurements of several disease at the household level. Oral health examination introduces much complexity to these global studies.

One alternative to professional clinical examinations are questionnaires that allow to obtain basic but relevant epidemiological information with lower costs [16]. The ENS 2016–2017 considers the application of questionnaires that include an oral health module and examination for people over 15 years. The health-related self-report has been used efficiently to evaluate disease such as cancer, rheumatoid arthritis, cardiovascular conditions as well as risk factors related to diet, physical activity and general health [17]. In dentistry, self-reporting is a valid instrument to study conditions such as oral hygiene, periodontal health and denture use [15, 16]. Studies conducted in the United States, Europe and Japan population suggest that the self-report of the number of teeth has been a tool of great validity; thus appropriate questions need to be included in the questionnaire [18].

Although there is available evidence on the validity of self-reports in determining the number of teeth in people’s mouths, the method needs to be validated in the Chilean population. Therefore, the aim of this study was to assess the validity of self-reports in estimating the number of teeth by using the findings of the dental examination as the gold standard.

## Methods

### Subjects

A random sample was selected from the participants of the ENS 2016–2017 who had undergone a complete dental examination. A stratified random sampling was carried out based on variables: sex (male/female), age (15–35, 36–60, 61 and over) and region in Chile (Metropolitan Region/other regions).

The sample size was estimated considering a two tailed test of comparison, a power of 80%, a value of statistical significance of 5%, an expected difference of 1.5 and a standard deviation of 3.93 teeth, according to previous similar studies in the literature [13]. The number obtained was 108 subjects, however, due to feasibility reasons during the implementation stage of the study, the final number was limited to 101 individuals.

As indicated in Table 1, in order to obtain 101 cases, we applied a sample oversize that considered a general loss of 50% within the universe of participants in the ENS 2016–2017. In this way, 202 cases were finally selected. Each participant was called at least once and three times at most, and codes were established to reflect the state of each call. Among the 202 eligible subjects, 137 participants were contacted, of whom 101 accepted to participate. The rejection rate of the study was 18% (36 subjects).

**Table 1:** State of cases. Oral health sub study, ENS 2016–2017

Contact	State	Cases	Distribution
Yes	Participate	101	50%
	Rejection	36	18%
No	Out of service	12	6%
	Busy/voicemail	8	4%
	No answer	45	22%
Total		202	100%

Since this data was collected by means of a telephone questionnaire, the subjects were required to meet the following additional eligibility criteria: i) registration of a valid and operational telephone number, which was recorded by a pollster or nurse during the field phase of the ENS 2016–2017 and ii) the ability to keep a coherent conversation by telephone (coherence refers to the way that participants cooperate to maintain a reasonably focused thread of conversation). In addition, violent subjects were excluded during the telephonic call.

### Dental examination

The oral health examination was performed as part of the ENS 2016–2017 by trained and calibrated nurses at home visits using a dental mirror, dental explorer and standard operation lamp. According to the pilot study of the ENS 2003 (*n* = 105 subjects), which evaluated the validity and reliability of the measurements taken by seven nurses against the diagnosis made by the dentist, sensitivity to detect missing teeth and dental fillings was 70%, when compared to the diagnosis by the dentist [11]. The inter-examiner reliability was substantial (kappa value of 0.75, *p*-value < 0,001) according to the criteria proposed by Landis and Koch [19].

In the ENS 2016–2017, nurses were trained by nine dentists that belong to the Ministry of Health of Chile. A theoretical presentation, a demonstration, an oral examination practice and a final test were carried out. The training was recorded by MINSAL TV to conduct an immediate feedback activity. During the oral examination demonstration, nurses were taught to use a standard operation lamp, a dental mirror, a dental explorer and a tongue depressor. In addition, the sequence of the oral examination was carefully explained and the records to be performed were reinforced. Regarding the oral examination practice, groups of two to three nurses were formed so that they could role-play the dental examiners. The final test comprised the evaluation of 20 clinical cases whose intraoral photographs were projected. With regard to these cases, 55 questions were asked about the topics evaluated in the oral health clinical examination of the ENS 2016–2017. The average observed score was 49.95 (SD 2.74) and a kappa coefficient of 0.85, *p* value < 0.01. The Nurses Manual and the Training Report for nurses who performed the oral examination of the ENS 2016–2017 are both available in the population survey repository of the Department of Epidemiology of the Ministry of Health of Chile: http://epi.minsal.cl/encuestas-poblacionales/. It should be noted that the ENS 2016–2017 Training Manual for nurses stated that during clinical examinations nurses must record the number of remaining teeth in both dental arches, without reading or informing the patients on evaluation of the registered numbers.

### Questionnaire

The telephone questionnaire was self-designed in Spanish and validated by an expert panel consisting of three dentists and two epidemiologists, incorporating two recurrent questions from similar previously published studies through the forward translation method [15, 20]. The telephone questionnaire was conducted by two interviewers belonging to the Centro UC Encuestas y Estudios Longitudinales (CEEL), both previously trained by a dentist. A telephone survey script was designed and delivered in a document to the two interviewers, which was rigorously used during the call. The survey consisted of three oral health questions with an average duration of seven minutes. First, the status of the call was completed (answered, not answered, busy, out of service, voicemail), then the participation status was recorded (participates/does not participate or rejects/re-call). Once the person agreed to participate, the following questions were asked: 1- “How many teeth do you have above?” 2- “How many teeth do you have below?” 3- “Have you had any tooth loss since the oral examination was performed by the ENS 2016-2017 nurse in your home?”. In case that the answer was “Yes”, they had to specify how many teeth were lost in that period. During the telephone call, subjects were asked to remove their dentures if they had any, and they could use a mirror to self-report the number of teeth, if necessary.

This study was nested in the NHS 2016–2017 whose protocols and written informed consent were approved by the Scientific Ethics Committee of the Faculty of Medicine of Pontificia Universidad Católica de Chile (CEC-MedUC, Project number 16–019). As to the telephone questionnaire, verbal informed consent was obtained from the participants after interviewers explained the purpose of the substudy.

### Statistical analysis

This study analysis was carried out in the crude subsample and it did not use the weights of the complex design of the main sample of the ENS 2016–2017. For the participants who reported tooth loss since the examination, the actual number of teeth was determined by subtracting the loss number. After this adjustment, the values obtained were compared to those indicated by the self-report.

The Spearman correlation coefficient was used to quantify the association between the self-report of the number of teeth and the record through the clinical examination. This analysis was performed for the total teeth in both dental arches, as well as for the total teeth in each dental arch of the subjects in the study, according to sex and age. Scatter plots were used to show self-reported number of teeth versus clinically determined number of teeth, where the points above the line indicated an overestimation and the points below indicated a lower notification of the number of teeth by self-report.

The Bland-Altman plot or difference plot was employed to evaluate the concordance between the two measurements of the number of teeth per subject. In this graphical method, the differences were plotted against the averages of the number of teeth obtained through clinical examination and self-report. Intraclass correlation coefficients (ICC) were calculated, and their interpretation was analogous to that of the kappa coefficient. Values lower than 0.4 reflected poor agreements, whereas ICC values above 0.75 indicated excellent concordance [19]. A statistical significance of 0.05 was established. The tests were conducted using the Statistical Package for Social Sciences (SPSS) version 24.0 (Mac OS X) software (SPSS Inc., Chicago, IL, USA).

## Results

Table 2 shows the characteristics of the individuals included in the study. The average age was 50 years for men and 51 years for women; 39.2% of men and 40% of women were older than 61 years old. In men, the average number of teeth in the examination (*n* = 22) coincided with the self-reported teeth average. In contrast, the average number of teeth reported (*n* = 19) in women was one unit higher than the average number of teeth observed during the clinical examination (*n* = 18).

**Table 2:** Characteristics of the study population, Oral health questionnaire validation sub study, ENS 2016–2017

	Men (*n* = 51)	Women (*n* = 50)	Total (*n* = 101)	*p*-value
Mean age (SD)	50 (18)	51 (19)	50,5 (18.4)	
15–35 years	23.5% (12)	26.0% (13)	24.8% (25)	
36–60 years	37.3% (19)	34.0% (17)	35.6% (36)	
> 61 years	39.2% (21)	40.0% (21)	39.6% (40)	
Number of teeth according to examination (mean/SD)	22 (8)	18 (11)	20 (10)	0.003*
Number of teeth according to self-report (mean/SD)	22 (9)	19 (11)	20 (10)	0.002*
Number of months since the examination (mean/SD)	5.35 (0.96)	5.18 (1.0)	5.27 (0.99)	*0.645*

*n* number, *SD* standard deviation. *: independent samples t test (*p*-value, a: 0,05)

In women, the mean number of teeth was significantly lower than the same value reported for men, in both the clinical examination (*p* = 0.003) and the telephone self-report (*p* = 0.002). For the total number of individuals, the average number of teeth obtained by both methods had the same value (*n* = 20). Since the examination was carried out by nurses the average elapsed time was 5.27 months (SD = 0.99) for the total number of individuals. During this period, 15 individuals lost teeth with a maximum of four losses. In subjects from the Metropolitan Region, the average number of teeth was 20 (SD = 8) and 20 (SD = 9) according to the examination and self-report respectively, while in other regions of the country the average number of teeth was 19 (SD = 9) and 19 (SD = 10) according to the examination and self-report respectively (not shown in Table 2).

**Fig. 1:**
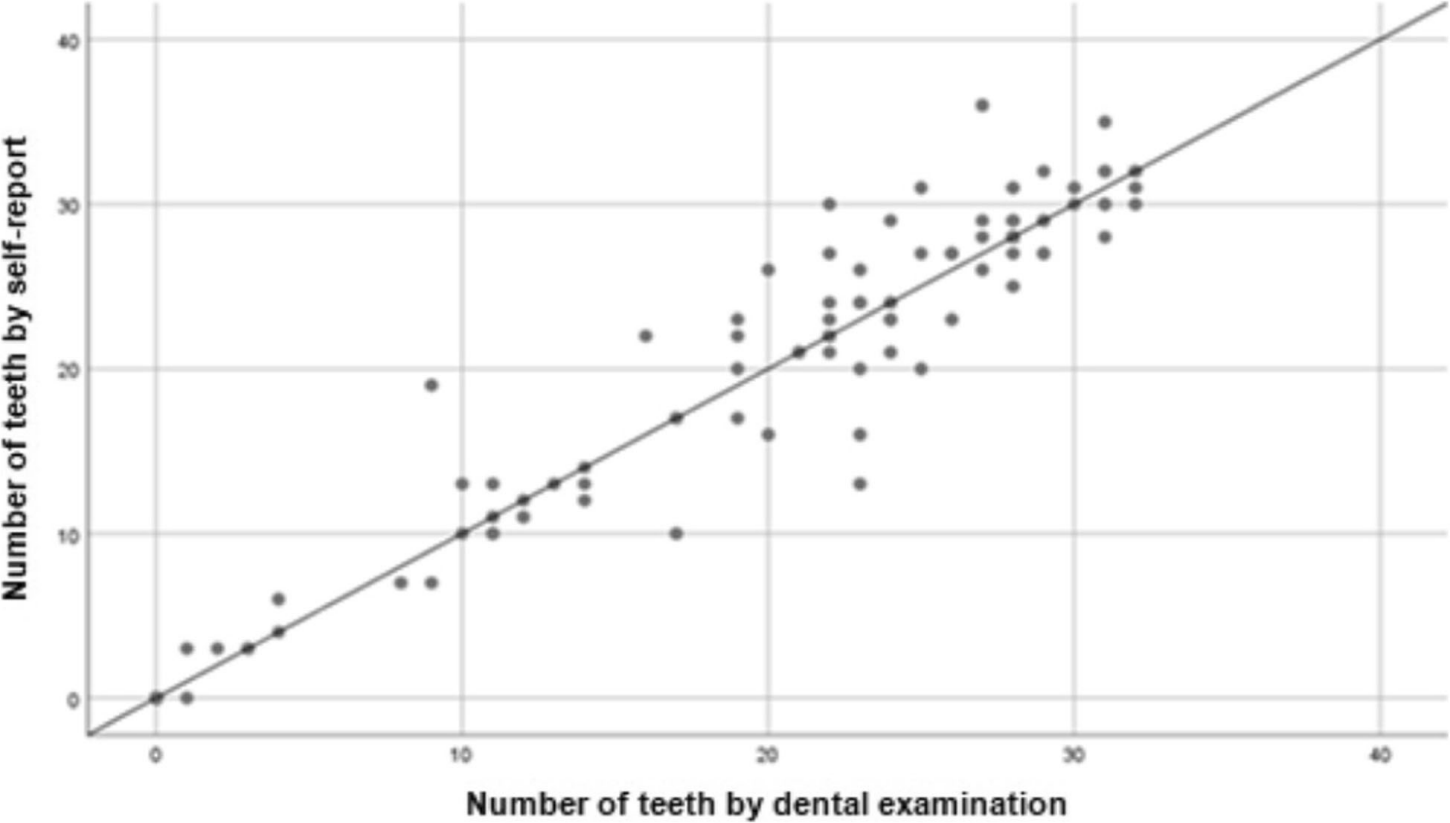
Association of self-reported and clinically-determined numbers of teeth

Table 3 shows the comparison between the number of teeth obtained in the dental examination during the ENS 2016–2017 and the self-report, according to the age and sex of the participants. In the total sample, a strong and significant correlation was obtained (Spearman ρ = 0.93, *p* < 0.01). In men, a high correlation was observed (Spearman ρ = 0.90, *p* < 0.01), becoming a trend that was maintained in each of the three age groups, whose coefficients were always above 0.75 (*p* < 0.01). In women, the correlation was also strong and significant (Spearman ρ = 0.96, *p* < 0.01) and the association observed increased significantly with age (*p* < 0.01). The value of the intraclass correlation coefficient indicated a very high agreement (ICC = 0.96, *p* < 0.01), in both men (ICC = 0.93, *p* < 0.01) and women (0.98, *p* < 0.01). Figure 1 shows a scatter plot of the number of teeth obtained by self-report relating to the number of teeth observed in the clinical examination for the total sample. The diagram indicates that the subjects accurately reported the total number of teeth during the telephone call.

**Table 3:** Comparison of the total number of teeth according to age, sex and type of measurement, ENS 2016–2017

	Total number of teeth (examination)		Total number of teeth (self-report)		ρ	*p*-value	ICC	*p*-value
	Median	Min/Max	Median	Min/Max				
Total (101)	23	0–32	23	0–36	0.93	< 0.01	0.96	< 0.01
Men								
Total (51)	24	0–32	23	0–32	0.90	< 0.01	0.93	< 0.01
15–35 years (12)	29	25–32	29	27–32	0.85	< 0.01	0.82	< 0.01
36–60 years (19)	24	12–32	24	12–31	0.85	< 0.01	0.86	< 0.01
> 61 years (20)	18	0–32	17	0–32	0.80	< 0.01	0.88	< 0.01
Women								
Total (50)	22	0–31	22	0–36	0.96	< 0.01	0.98	< 0.01
15–35 years (13)	28	20–31	28	26–35	0.85	< 0.01	0.63	< 0.01
36–60 years (17)	23	0–31	22	0–36	0.92	< 0.01	0.96	< 0.01
> 61 years (20)	11	0–26	11	0–24	0.97	< 0.01	0.98	< 0.01

*Min* minimum, *Max* maximum, *ρ* Spearman’s correlation coefficient, *ICC* intraclass correlation coefficient

**Fig. 2:**
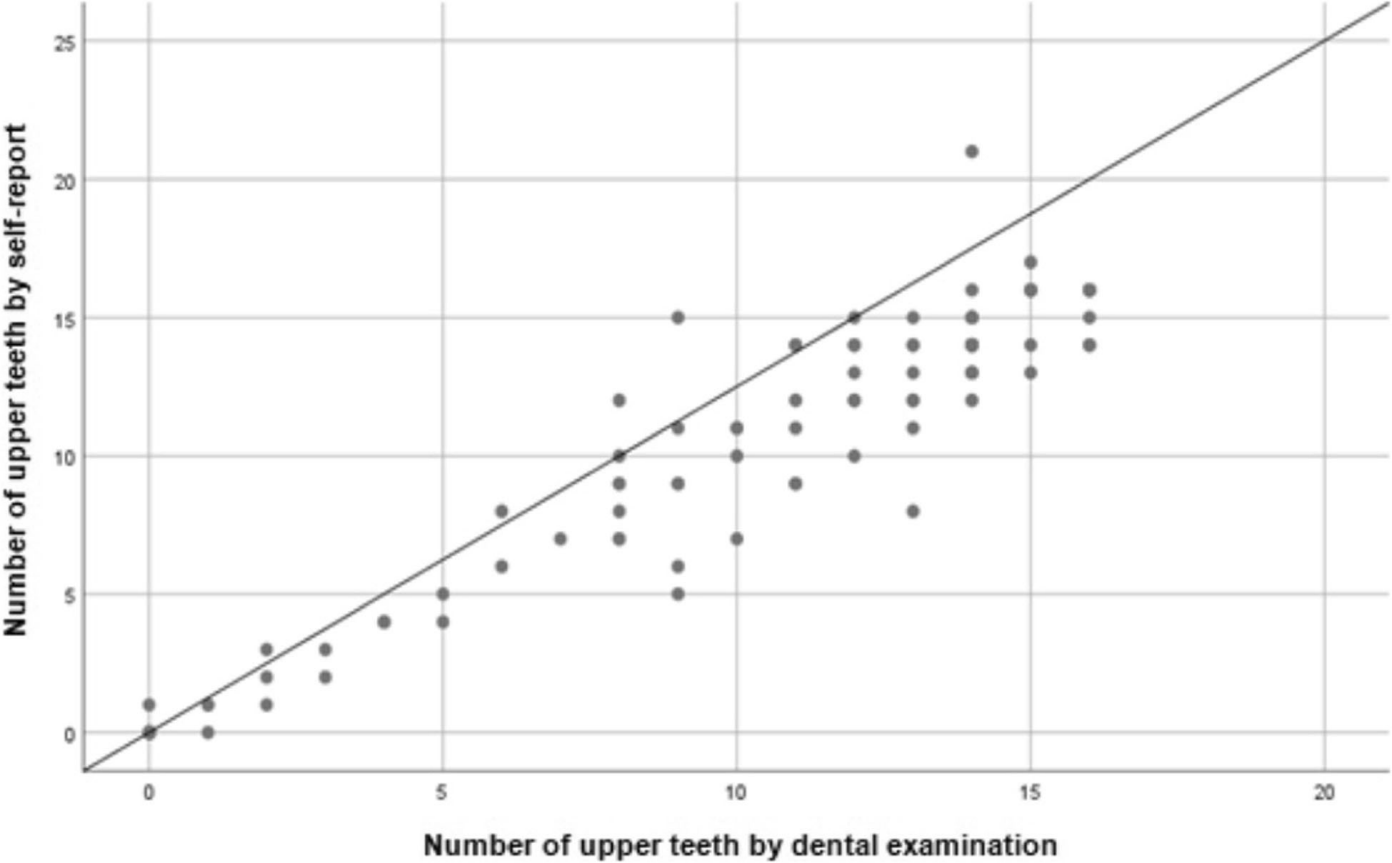
Association of self-reported and clinically-determined numbers of upper teeth

Table 4 contains a comparison between the number of upper teeth observed in the dental examination and the telephone self-report, according to the age and sex of the participants. A strong and significant correlation was obtained (Spearman ρ = 0.91, *p* < 0.01) for the number of upper teeth. In men, a strong and significant correlation was obtained (Spearman ρ = 0.88, *p* < 0.01), then a similar situation occurred in women (Spearman ρ = 0.95, *p* < 0.01). In both sexes, the value of the Spearman coefficient increased with age. The value of the intraclass correlation coefficient indicated a very high and significant agreement (ICC = 0.96, *p* < 0.01), in both men (ICC = 0.94, *p* < 0.01) and women (ICC = 0.97, *p* < 0.01). Figure 2 shows the scatter plot for the number of upper teeth in the mouth.

**Table 4:** Comparison of the number of upper teeth according to age, sex and type of measurement, ENS 2016–2017

	Total number of teeth (examination)		Total number of teeth (self-report)		ρ	*p*-value	ICC	*p*-value
	Median	Min/Max	Median	Min/Max				
Total (101)	11	0–16	12	0–21	0.91	< 0.01	0.96	< 0.01
Men								
Total (51)	12	0–16	12	0–16	0.88	< 0.01	0.94	< 0.01
15–35 years (12)	14	12–16	14	13–16	0.55	0.06	0.53	0.03
36–60 years (19)	13	3–16	12	3–16	0.81	< 0.01	0.91	< 0.01
> 61 years (20)	6	0–16	8	0–16	0.87	< 0.01	0.90	< 0.01
Women								
Total (50)	10	0–16	11	0–21	0.95	< 0.01	0.97	0.01
15–35 years (13)	14	11–16	14	13–17	0.78	0.002	0.59	0.01
36–60 years (17)	10	0–6	11	0–21	0.93	< 0.01	0.94	< 0.01
> 61 years (20)	2	0–15	2	0–16	0.98	< 0.01	0.97	< 0.01

*Min* minimum, *Max* maximum ρ Spearman’s correlation coefficient, *ICC* intraclass correlation coefficient

**Fig. 3:**
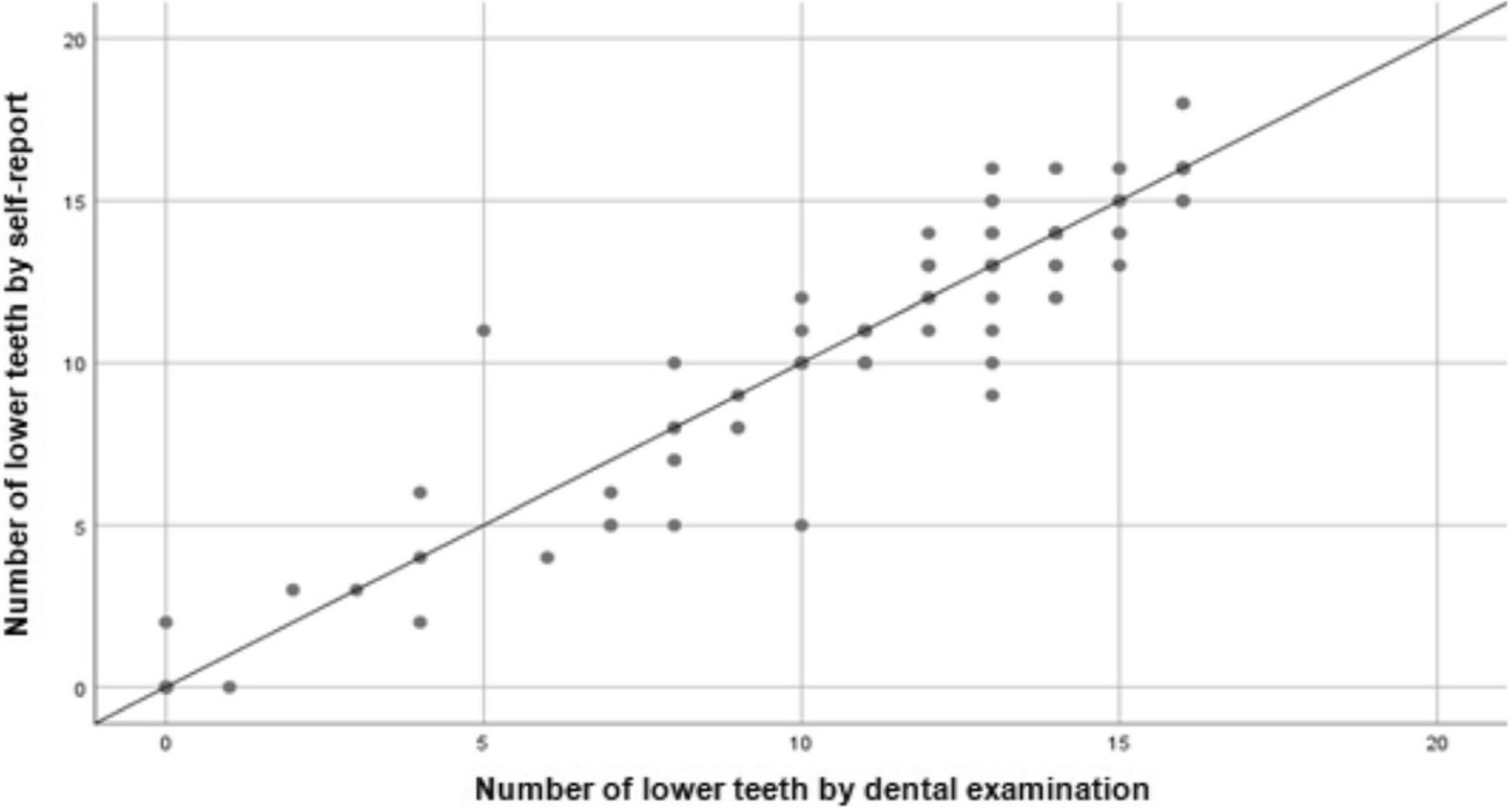
Association of self-reported and clinically-determined numbers of lower teeth

Table 5 contains a comparison between the number of lower teeth, according to the age and sex of the participants. The correlation was strong and significant (Spearman ρ = 0.93, *p* < 0.01) for the number of lower teeth. In men, a strong and significant correlation was obtained (Spearman ρ = 0.91, p < 0.01) but it was still lower than the one observed in women (Spearman ρ = 0.95, p < 0.01). The value of the intraclass correlation coefficient indicated a very high and significant concordance for the lower teeth (ICC = 0.96, *p* < 0.01), which was mirrored in men (ICC = 0.91, *p* < 0.01) and women (ICC = 0.98, *p* < 0.01). Figure 3 shows the scatter plot for the number of lower teeth in the mouth.

**Table 5:** Comparison of the number of lower teeth according to age, sex and type of measurement, ENS 2016–2017

	Total number of teeth (examination)		Total number of teeth (self-report)		ρ	*p*-value	ICC	*p*-value
	Median	Min/Max	Median	Min/Max				
Total (101)	12	0–16	12	0–18	0.93	< 0.01	0.96	< 0.01
Men								
Total (51)	13	0–16	12	0–16	0.91	< 0.01	0.91	< 0.01
15–35 years (12)	15	13–16	14	13–16	0.92	0.06	0.92	< 0.01
36–60 years (19)	13	8–16	12	8–16	0.83	< 0.01	0.83	< 0.01
> 61 years (20)	11	0–16	11	0–16	0.86	< 0.01	0.88	< 0.01
Women								
Total (50)	12	0–16	11	0–18	0.95	< 0.01	0.98	< 0.01
15–35 years (13)	14	12–16	14	13–18	0.70	0.004	0.74	< 0.01
36–60 years (17)	13	0–16	13	0–16	0.90	< 0.01	0.96	< 0.01
> 61 years (20)	8	0–14	7	0–13	0.98	< 0.01	0.97	< 0.01

*Min* minimum, *Max* maximum ρ Spearman’s correlation coefficient, *ICC* intraclass correlation coefficient

**Fig. 4:**
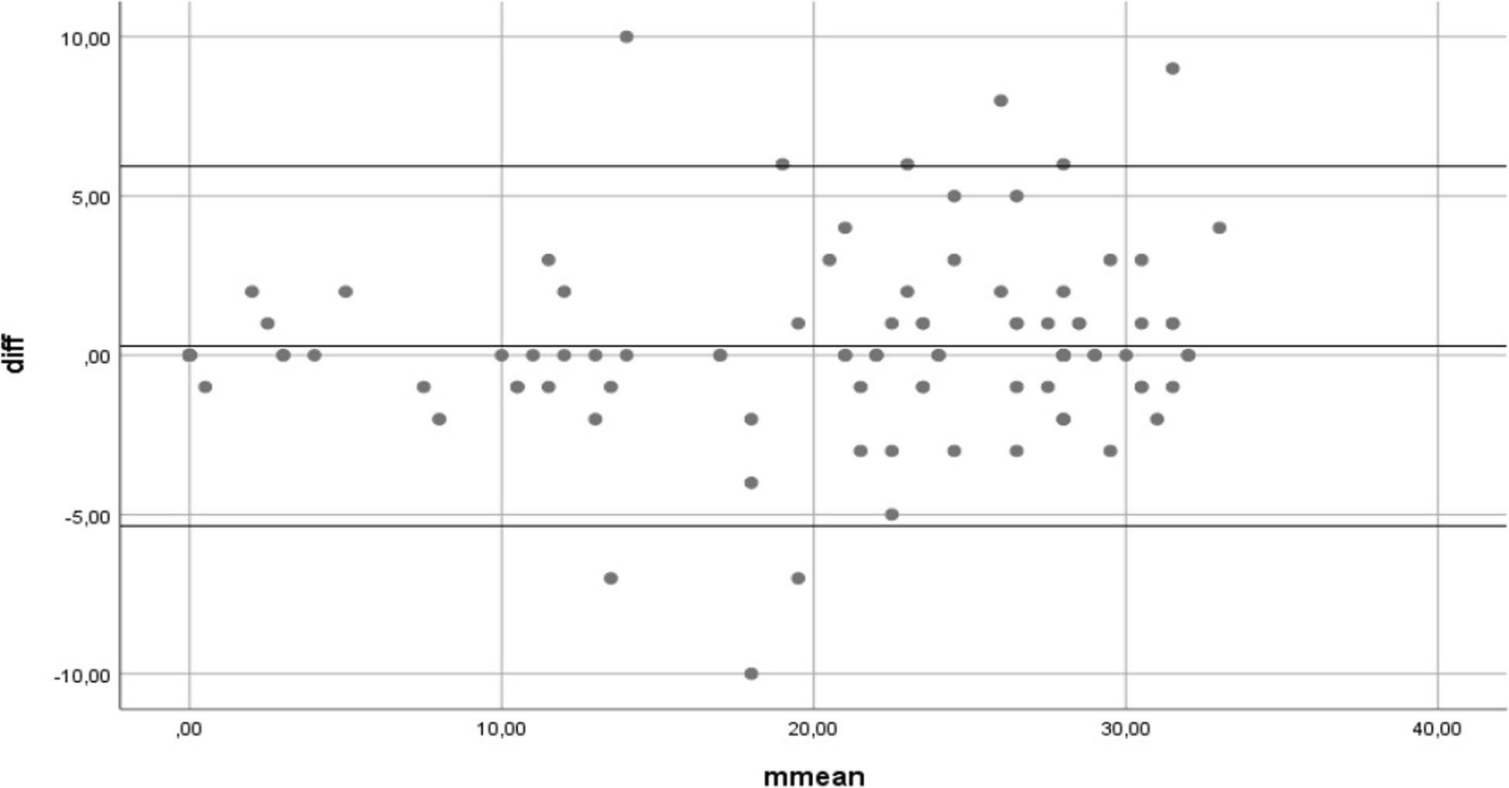
Agreement of self-reported and clinically-determined numbers of teeth. “Bland-Altman method (*n*=101). Axis of the abscissa mean average of the total number of teeth according to the self-report and clinical examination. Axis of the ordinates, diff: difference of the number of teeth according to the self-report and clinical examination. *SPSS 24.0”*

Figure 4 shows a chart corresponding to the Bland-Altman method that evaluates agreement on the determination of the total number of teeth by the two measurements. A mean of the difference between both methods of 0.29 (SD = 2.88; *p* = 0.319; 95% CI = − 0.2818, 0.8561) was obtained. There was not a significant systematic difference between both methods because the line of equality (y = 0) was within the confidence interval of the mean difference. The diagram shows that most differences were approximately between the mean of the difference and two standard deviations, which indicates that the differences were normally distributed. Based on the Bland-Altman method, we quantified an acceptable range of agreement between − 5,4 and + 5,9. In this regard, the smaller the total number of teeth reported by a subject was, the closer the points were to the line of agreement. The Bland-Altman method showed concordance between the measurements of the total number of teeth by self-report compared to the clinical examination.

## Discussion

The number of teeth self-reported by the subjects in this study correlated significantly with the number of teeth obtained in the clinical examination, in both men and women. The mean number of teeth determined by self-report coincided with the one calculated by the clinical examination. Moreover, there was a marked and significant agreement between the measurements obtained by both methods, suggesting that self-reports are as valid an instrument to determine the total number of teeth a person has as the clinical examination performed by trained nurses, an instrument that was used as an epidemiological surveillance indicator in the ENS 2003 and the ENS 2016–2017.

In this study, the obtained correlation coefficient (Spearman ρ = 0.93) had a higher value than others previously published in the literature. Ueno et al. [13] reported a Pearson coefficient of 0.80 and an intraclass correlation coefficient of 0.78 for all participants in 1152 Japanese subjects from 40 to 56 years of age. Also, in Japanese adults, Matsui et al. [15] observed a Spearman correlation of 0.69, when analysing 1501 subjects. Our intraclass correlation coefficients also exceeded those reported by Gregg et al. who determined values of 0.87 for the total teeth, 0.89 for upper teeth and 0.78 for lower teeth in black and non-Hispanic white American individuals [21].

These high coefficients may be related to the fact that in our study the telephone questionnaire gave the subject additional instructions such as “counting or using a mirror” before providing an answer. On the other hand, the term “natural teeth” was not considered in the questions, which has led to the exclusion of teeth abutting a crown and bridge, and therefore reporting a lower number of teeth than the one determined during the clinical examination [15]. Patients were also asked to remove any dentures when reporting the number of teeth in order to prevent prosthetic teeth from being included in the report, thus improving data accuracy.

Compared to our findings, other authors have shown results with higher coefficients for the total number of teeth. Douglass et al. reported a correlation of 0.97, but they only included 50 individuals, all them aged over 70 years, whereas the self-report was facilitated by a lower mean number of teeth in mouth decreasing the differences with the clinical examination measurement [20]. The authors specified a correlation of 0.95 for upper teeth and of 0.98 for lower teeth. Although these values are higher than our coefficients, they show a similar tendency for greater precision in the self-report of lower teeth [20].

The intraclass coefficients were lower in subjects between 15 and 35 years old, particularly in women, regarding the upper and lower teeth comparisons. The values of agreement between the studied methods increased with age, particularly in women. This situation has been connected to the idea that more adults have a greater knowledge of their own oral health [22]. On the other hand, the variability in the total number of teeth affects the self-report [20]. Ueno et al. showed that the intraclass correlation coefficient in people with 1 to 19 teeth was 0.72, while in subjects with 20 to 32 teeth the value decreased to 0.62, a trend consistent with our present analysis [13]. Similarly, the effect on the correlation coefficients of variables age and sex requires an adjusted analysis by educational level, which was not performed in this study.

An advantage of our study was that the questionnaire design included WHO recommendations for population surveys in dentistry, specifically those about the use of a simple, short structure and at the same time formed with valid questions [23]. The application of this questionnaire has lower costs compared to clinical examinations and allows important information on oral health morbidity to be obtained [15, 24]. However, as to the limitations of this study, we recognized the low sample size and the fact that we did not explore other variables such as the presence of plural fixed prostheses, dental implants, retained root fragments and supernumerary teeth, conditions that can produce measurement biases. For example, Gregg et al., found different correlation coefficients, depending on the number of retained root fragments in the mouth: 0.88 for subjects without retained root fragments, 0.77 for subjects with only one retained root fragment, 0.68 for subjects with two retained root fragments and 0.87 for subjects with three or more retained root fragments [21]. Another limitation of this study refers to the eruption of wisdom teeth in individuals between 17 and 21 years old which could have influenced the difference in the number of self-reported teeth, especially considering that 24.8% of the sample was between 15 and 35 years old.

An additional limitation of the present study is that the questionnaire was administered to subjects that were previously examined by nurses in the ENS 2016–2017 and may have had greater knowledge of their oral health. Nevertheless, the nurses were trained to not reveal the tooth count result to participants and only to type it into the electronic recording device. In addition, the prospective extrapolation of the number of teeth, using the self-report of tooth losses since the nurse visit, may be affected by memory biases of the interviewees.

Finally, the validity of the self-report of the number of teeth in Chilean adults should continue to be investigated. By comparing this information with the clinical examinations performed by dentists, a method that is considered the *gold standard,* the qualities of dental self-reports could be supported as a diagnostic test. Also, future investigations about this topic should consider a higher sample size to confirm the findings of the present study.

## Conclusions

Despite the existing limitations, the findings from this study show that at the population level it is feasible to implement self-reports as a valid substitute to clinical examinations performed by trained nurses, to evaluate the number of teeth that an individual has. This form of measurement is a simple and inexpensive tool that provides useful information for household studies which focus on epidemiological surveillance of simultaneously occurring chronic diseases in the general adult population.

## Data Availability

The datasets generated and analysed during the current study are available in the Population Survey repository of the Department of Epidemiology of the Ministry of Health of the Government of Chile, http://epi.minsal.cl/encuestas-poblacionales/

## Abbreviations

DMAs: Dento-Maxillary Anomalies
ENS: National Health Survey
MINSAL: *Ministerio de Salud*, Ministry of Health
WHO: World Health Organization
